# Development of an eHealth Intervention in Pediatric Home Infusion Therapy: Interview Study of Needs and Preferences of Parents and Health Care Professionals

**DOI:** 10.2196/63260

**Published:** 2025-03-13

**Authors:** Helena Hansson, Charlotte Castor, Hanne Bækgaard Larsen, Martha Krogh Topperzer, Mette Linnet Olesen

**Affiliations:** 1 Department of Paediatrics and Adolescent Medicine, Copenhagen University Hospital Rigshospitalet, Blegdamsvej 9, Copenhagen, 2100, Denmark, 45 35459400; 2 Department of Clinical Medicine, University of Copenhagen, Copenhagen, Denmark; 3 Department of Health Sciences, Lund University, Lund, Sweden; 4 The Paediatric Oncology Research Laboratory, Copenhagen University Hospital Rigshospitalet, Copenhagen, Denmark; 5 Department of Education, Copenhagen University Hospital Rigshospitalet, Copenhagen, Denmark; 6 Department of Gynaecology, Fertility and Obstetrics, Copenhagen University Hospital Rigshospitalet, Copenhagen, Denmark

**Keywords:** pediatrics, digital, interventions, eHealth, home care, intravenous infusion, qualitative research

## Abstract

**Background:**

With the provision of home infusion therapy in children with acute or long-term illness on the rise, eHealth technologies have the potential to bridge the transition between hospital and home. However, eHealth interventions intended to support parents in managing home infusion therapy are sparse. Gaining insight into the needs and experiences of parents and health care professionals is crucial to developing feasible and sustainable eHealth interventions that target their needs. This study describes the first phase of a research study designed to develop and evaluate an eHealth intervention to support home infusion therapy.

**Objective:**

This study aimed to identify the experiences and needs of parents and health care professionals during home infusion therapy and their preferences for digital features in a future eHealth intervention.

**Methods:**

A qualitative study was conducted at 3 pediatric departments at a university hospital in Denmark. We individually interviewed 17 parents of 14 children who had received home infusion therapy with a portable pump. In addition, 5 focus groups were conducted with 15 health care professionals. We conducted a qualitative content analysis of the data, which we collected from February to July 2020.

**Results:**

We identified 6 subthemes that we merged into 3 main themes: increasing safe self-management at home; adapting information and responsibility to individual changing needs; and requesting digital features to ensure skill level, safety, and quality of care. The analysis showed that parents and health care professionals had corresponding needs and preferences, for example, a need for a high sense of safety and easier ways to communicate during home infusion therapy. Both groups emphasized the need for digital features to improve problem-solving and communication as a supplement to existing care to promote a safe environment, self-management, and quality of care. A vital issue was that an eHealth intervention should be aligned with the workflow of health care professionals and comply with regulations regarding confidentiality in communication and data sharing.

**Conclusions:**

Our study highlights the needs that parents and health care professionals have for increased safety and easier access to communication when receiving and providing home infusion therapy. The findings will be used to help develop an eHealth intervention supporting home infusion therapy tailored to individual needs.

## Introduction

Home infusion therapy for children with acute or long-term illnesses is becoming increasingly common due to its benefits, such as supporting children and their families in maintaining their everyday lives and improving the child’s health outcomes [1]. This therapy can be provided by nurses in hospitals or municipalities, home care agencies, or by nonprofessional caregivers such as parents or guardians. Transitioning care tasks from a hospital to a home setting is complex and requires well-functioning, coordinated collaboration between health care professionals and parents [2]. Parents have reported feeling anxious and insecure if they lack support, experience with, or knowledge about care tasks such as administering medications and observing symptoms that they must manage at the hospital and at home [3]. Furthermore, studies show that parents’ administration of oral medication at home is a high-risk area for medication errors [4-7]. Thus, it is vital to ensure that parents are comfortable and confident when managing care tasks at home.

eHealth interventions can be a means to support parents since they provide digital resources for managing home infusion therapy. According to the World Health Organization [8], eHealth can increase communication and data sharing between health care professionals and patients and improve accessibility and patient participation by electronic technologies. The use of eHealth expanded radically during the COVID-19 pandemic [9], but various barriers remain before integration into pediatric clinical practice is completely successful [10-13]. Studies have reported that the use of eHealth in pediatric clinical care can bridge the transition from hospital to home and increase interaction between the two by efficiently and conveniently providing information and communication [14-16]. This helps promote confident self-management among parents in treating and caring for children with various medical needs at home [2,15-17], improve symptom control in the child, and reduce parental worries and insecurity [2,18-22]. However, the availability of eHealth interventions for families of children with acute or long-term health conditions as well as the evidence supporting eHealth in pediatric clinical practice, remains still limited [23-25].

At the outset of this study, children and adolescents were given the opportunity to start antibiotic infusion therapy using a portable pump at a university hospital in Denmark and then continue treatment at home. Parents were trained by nurses and supported with printed materials and resources on the hospital’s website. The provision of home infusion therapy began to expand to include other types of medications and delivery methods, thereby placing greater responsibility on the parents. No eHealth interventions were available to support home infusion therapy and there was uncertainty about the extent of parents’ and health care professionals’ needs and the specific content that they may need for eHealth support. Previous research have shown that extensive support is essential when parents perform caregiving tasks at home [3,26]. However, less is known about how parents and health care professionals employed at hospitals perceive their needs and preferences related to home infusion therapy and eHealth support. According to Medical Research Council’s framework [27], exploring the needs of intervention users is a crucial first step in developing an intervention. Applying a participatory design [28], future users are involved to gain an understanding of their situation and respond to their experiences, needs, and preferences. This approach ensures that a future eHealth intervention would target their needs and be sustainably implemented [11,28-30]. Therefore, the aim of this study was to identify the experiences and needs of parents and health care professionals regarding home infusion therapy, in addition to their preferences for including digital features in the development of a future eHealth intervention.

## Methods

### Design

An exploratory qualitative design was applied to get relevant input from the users to the future development of a supportive eHealth intervention. This study is a part of the developmental phase of a larger research program with the overall goal of developing and testing an eHealth intervention to support parents and health care professionals during pediatric home infusion therapy. The Medical Research Council’s framework for the development and evaluation of complex interventions [27] and a participatory design [28] framed the overall research project. The framework guides researchers through 4 interconnected phases, development, feasibility, evaluation, and implementation. Engaging the users of the eHealth intervention throughout the research study ensured the developmental relevance of our study and its feasibility in clinical practice, improving the probability of successful implementation [26].

### Setting

The study was conducted in 3 pediatric departments at a university hospital in Copenhagen, Denmark, 1 specializing in oncology and hematology (20 in-patient beds), 1 in organ and infection diseases (12 in-patient beds), and 1 day hospital (7 beds), with around 2000 combined admissions annually.

### Home Infusion Therapy

In 2018, the departments started offering home infusion therapy with portable pumps as an option to children with diagnoses such as cancer or with an acute infection with no underlying condition. The therapy was primarily used for intravenous antibiotic treatment and always initiated during hospitalization at an in-patient ward or in an out-patient setting. The children had a central venous catheter or midline catheter. A nurse informed and trained primarily one of the child’s parents before home infusion therapy was initiated and administered the portable pump to the child at the hospital before discharge. The child returned the following day to the hospital to receive a new dose in the pump or to discontinue treatment. The parent’s task was to clinically observe the child, the pump and infusion at home, and react if problems arose, such as reactions to the medicine or a pump alarm sounding. If the child had a serious reaction, the parent was instructed to immediately call an ambulance. Health care professionals also taught some parents how to change the pump medication or replace the elastomeric pump with a new one to allow the family to stay at home 1 or 2 additional days before returning to the hospital,

### Participants

A purposeful sampling strategy of children, parents, and health care professionals was used to obtain rich and varied data on the home infusion therapy [31]. Inclusion criteria for children were 0 to 18 years of age, having received home infusion therapy with a portable pump with any type of medication, having the parent living with the child part or full time, and the parent speaking Danish or English. Most children receiving the therapy had an underlying long-term illness, but children with an acute infection with no underlying condition were also approached. In total, 14 children and their parents were informed about the study, and all agreed to participate. The children were invited to participate in an interview together with their parent, which resulted in 13 children participating, 2 of whom actively joined in. In 3 families, both parents participated and were interviewed jointly or separately (Table 1). Inclusion criteria for health care professionals were: (1) nurse or a physician, (2) working at one of the 3 departments, and (3) had experience with providing home infusion therapy. A total of 15 health care professionals were approached, and all agreed to participate.

**Table 1.** Demographic and clinical characteristics of children and parents.

Characteristic			Participant, n
Children (n=14)			
	Age (years)		
		0‐5	5
		6‐11	6
		12‐14	1
		15‐18	2
	Sex		
		Male	11
		Female	3
	Primary diagnosis		
		Cancer	10
		Congenital lung disease	2
		Congenital autosomal disorder	1
		Stroke caused by virus infection	1
	Ethnicity		
		Danish	11
		Other	3
	Cohabiting with siblings (n)		
		0	2
		1	9
		2‐4	3
Parents (n=17)			
	Sex		
		Female	12
		Male	5
	Partner relations		
		Cohabiting with partner	16
		Single parent	1
	Age (years)		
		31‐40	11
		41‐50	3
		Unknown	3
	Occupational status		
		Employed	5
		Unemployed	1
		Paid leave due to child’s illness full time	10
		Paid leave due to child’s illness part time	1

### Data Collection

Data were collected from February to July 2020. Parents were interviewed based on a 2-part exploratory semistructured interview guide. The first part covered experiences and needs regarding home infusion therapy in terms of provision of and responsibility for it, preparation and training, and sense of safety, worries, and challenges. The second part presented concrete examples of digital features to generate ideas on how technology could address the needs and preferences of the child and parent, in addition to focusing on how an eHealth intervention could support home infusion therapy. We asked which digital features to include, such as possible advantages and disadvantages, experience with digital technologies, and what features are important to have in an eHealth intervention. The eHealth Literacy Framework provided the underlying inspiration for the questions in the second part to understand user needs and prerequisites when new digital health services are introduced [32]. The interviews were conducted by the first author (n=16), at one of the pediatric departments in a meeting room or an in-patient room lasting and lasted 30 to 120 minutes, though 1 was done by phone.

Health care professionals were interviewed in 5 separate focus groups with a semistructured interview guide corresponding to the guide for parents though tailored to their professional role. The focus group interviews, which lasted 60 to 90 minutes, were held in a meeting room at one of the pediatric departments. The interviews were recorded and transcribed verbatim.

### Data Analysis

A qualitative content analysis of the transcribed interviews was used comprising a 5-step iterative and inductive approach based on Graneheim and Lundman [33] to identify any patterns and variations in participant experiences. First, 2 authors [HH and MLO] read the text independently to gain an overall understanding. Second, they identified meaning units in terms of words, sentences, and paragraphs that related to the study aim. Next, the meaning units were condensed, organized, and coded using NVivo software (Lumivero). Fourth, the codes were compared with identify similarities and differences, before being organized into subthemes. Finally, the subthemes were compared and analyzed, after which they were merged into main themes. The defined main themes had a consistent pattern of underlying meaning in terms of the condensed meaning units, codes, and subthemes. To strengthen the trustworthiness of the study, the same 2 authors discussed the meaning units, codes, subthemes, and themes throughout the analysis until reaching consensus. Initially, individual and focus group interviews were analyzed separately as 2 datasets. During the data analysis, it became apparent that the subthemes overlapped, and data were analyzed together. In the last phase of the analysis, all authors reflected on and discussed the main themes and subthemes.

### Trustworthiness

The authors discussed the analysis, interpretations, and themes at seminars and presentations with key health care professionals involved in home infusion therapy and other researchers to enhance credibility. The authors also discussed their preunderstanding to consider how it may influence data collection and analysis, all of us are experienced in conducting qualitative studies. The first author [HH], who is a nurse, has experience with implementing home infusion therapy at the 3 departments but did not have any clinical contact with the families before the interviews. The health care professionals knew HH, which may have hampered them from freely expressing themselves, but her knowledge of home infusion therapy may also have made them feel confident enough to provide in-depth responses to the interviews.

### Ethical Considerations

The Danish Protection Agency approved the study (P-2019‐392), while approval from the Regional Research Ethics Committees for the Capitol Region of Denmark was not required since it only assesses studies that collect biological material. The study adhered to the Declaration of Helsinki, International Ethical Guidelines for Health-Related Research Involving Humans, and current Swedish and European law. All participants were assured that participation was voluntary and that they could withdraw from the study at any time without affecting their child’s treatment or the health care professionals’ work. All participants provided written informed consent and were assured confidentiality. When applicable, the children received age-appropriate information about the study based on their cognitive abilities, language skills, and legal guardian’s preferences.

## Results

### Overview

The amount of experience parents and health care professionals had with home infusion therapy ranged from doing it a few to multiple times. The parents expressed that it enhanced their child’s well-being and the whole family’s due to having a greater opportunity to spend everyday life together more at home. They were willing to take on the additional caregiving tasks and responsibility due to the benefits associated with doing so. The health care professionals also described how the child and family benefited and how important the option of home infusion therapy was.

Three main themes were identified comprising six subthemes: (1) increasing safe self-management at home, (2) adapting information and responsibility to individual changing needs, and (3) requesting digital features to ensure skill level, safety, and quality of care (Textbox 1). The themes and subthemes were bound together in an overarching theme “managing the extended responsibility of home infusion therapy.” This theme encompasses the core experience of the increased responsibility parents take on when managing the therapy and the health care professionals being responsible for therapy taking place outside the hospital. Digital features were described as means to support that responsibility.

**Figure 1.**
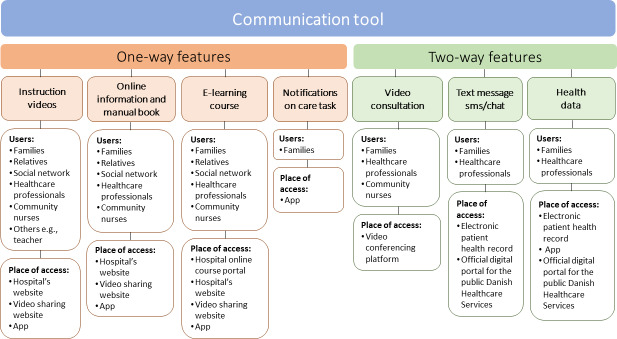
Digital features proposed by parents and health care professionals for a future eHealth intervention.

The first and second themes describe the needs of parents and health care professionals based on current experiences with home infusion therapy, while the third main theme present the digital features proposed to meet the needs of both groups in a future eHealth intervention (Figure 1).

Textbox 1. **Main themes and subthemes.**

**Overarching theme:**

- Managing the extended responsibility of home infusion therapy

**Main themes and their subthemes:**

- Increasing safe self-management at home
  - Need for standardized information, training, and shared problem-solving
  - Being in control of home infusion therapy and feeling safe

- Adapting information and responsibility to suit individual needs
  - Repeating individualized information and training
  - Desire to share the responsibility

- Requesting digital features to ensure skill level, safety, and quality of care
  - Access to online one-way communication of knowledge and training
  - Access to interactive communication and support

### Theme 1. Increasing Safe Self-Management at Home

#### Subtheme: Need for Standardized Information, Training, and Shared Problem-Solving

Overall, parents described that they were confident about the information and training they had received on how to manage home infusion therapy, and they had not experienced any problems with the pump or the child’s health. But some felt that the information and training were too sparse. They appreciated the information booklet, even though they did not always have it with them when they needed it. The hospital website provided information and guidelines, but finding either one was sometimes difficult and time-consuming if immediate support was required.

Some parents thought that the information and training they received was inconsistent, making both them and their child feel insecure. They requested uniform guidelines and training regardless of which health care professionals or department taught them about managing the care tasks. They also felt unsure if they called for assistance and spoke with a health care professional who lacked the skills to provide support, sometimes forcing families to travel to the hospital.

*I felt stressed when it [pump] sounded an alarm that showed ‘low reservoir volume’. I think it was actually when the infusion bag was full. And then I called the ward after trying everything I could do at home. But when you call in the evening, there isn’t necessarily anyone at work who knows much about the pump. So, they didn’t know what I should do either*.

[Mother 232]

Health care professionals who seldom had the task of giving parents information and training about managing the portable pump worried about how to maintain their professional skill level, making them feel insecure professionally and concerned about how that could affect the children and parents.

*We don’t provide home infusion that much at our ward and the more you physically deal with it [pump] in your hands, the better, safer, and more familiar you get with it when you teach the parents*.

[Health care professional 113]

#### Subtheme: Being in Control of Home Infusion Therapy and Feeling Safe

The parents described that the pump was easy to manage and that they did not experience many problems related to the pump and infusion, or that their child experienced adverse effects. If a pump alarm went off, they were mostly able to manage the issue at home. They felt particularly worried if an alarm sounded due to air bubbles in the infusion set. In such situations they expressed a need for different interaction than a telephone call with the health care professionals because it had the potential to resolve problems and thereby avoiding a hospital visit.

*I think, I think that there’s a need for extra support in those kinds of situations when it [pump] acts up*.

[Mother 73]

Parents described how they wanted to take on responsibility for their child’s home infusion therapy as it was a way to have control over their situation. Even though they generally felt secure about the information, training, and support they received, some parents still worried about something unexpected happening, making them feel vulnerable about being home alone without a health care professional nearby. They stated that they would appreciate more support to improve their sense of safety and control of the situation.

The health care professionals also described the need to have more control when discharging a child to home infusion therapy, mentioning that they felt an extended sense of responsibility toward the child and home treatment that they sometimes found difficult to fulfill since they were unable to monitor the child’s condition in the same way as at the hospital. The health care professionals lacked having a sense of the family’s capacity at home; for example, they could not assess whether the procedures and observations the parents took on had been performed correctly. This lack of control preoccupied them as they worried about the risk of medication errors and adverse events. The health care professionals needed reassurance that the parents had conducted the necessary observations and procedures to help increase their feeling of control concerning the treatment they had initiated, especially because they felt responsible for the child’s well-being, treatment, observations, and transferring such specialized care tasks to the parents without their presence to safeguard them at home.

*And then there’s the issue of whose responsibility it is when you hand it [therapy] over to the parents? Because I agree that they only want the best for their child. There’s no doubt about that*.

[Health care professional 117]

### Theme 2. Adapting Information and Responsibility to Suit Individual Needs

#### Subtheme: Repeating Individualized Information and Training

Parents described feeling mentally burdened by their child’s illness, especially those whose child had long-term illness. They had difficulty remembering and comprehending information and training, causing them to request the possibility to repeat information and training once they returned home.

*You’re bombarded with things that I, as a parent, must be able to gain an overview of, including how the pump works. Then, when you get home you think: “God, what was it that they said about that?” And in that situation, it would be really nice if you could seek help [digitally/online]: “Could you tell me again, what I should do, when I should do this and that?”*

[Mother 173]

The health care professionals also worried that the parents did not always have the mental surplus to handle new or additional tasks in relation to home infusion therapy. They expressed a need for providing more individualized support to parents at home as they saw the parents’ mental surplus as changing over time. It was important for them to ensure that the parents felt safe and were not additionally burdened by managing home infusion therapy.

*Maybe we could come by to force them to accept it [home infusion], because we’ve been used to that. Then there’s a psychological aspect to it, where you constantly have to determine where they are in the treatment trajectory: “Do you still feel secure about doing home care?” Because I think the families experience a tremendous number of mental ups and downs*.

[Health care professional 262]

#### Subtheme: Desire to Share the Responsibility

The parents described that they would like to involve other caregivers and professionals in the home infusion therapy to share the responsibility, also their child if the child wanted to be involved. Often only one parent was trained to manage the pump and medicine, leaving them with the sole responsibility. One parent said that her partner did not want to help with it due to how unsure it made him feel.

*He’s perhaps afraid of ending up doing something wrong [with the pump] that may harm our son, which I think might paralyze him a little in terms of his ability to act*.

[Mother 196]

Another parent described how the partner, who had not been trained at the hospital, panicked at home when an alarm on the pump went off. Parents also emphasized the need for additional support to facilitate training and guidance at home to increase the sense of safety and control for the child and other caregivers with the therapy and to support shared responsibility. Like the parents, the health care professionals supported and emphasized the need for parents to share the responsibility for managing home infusion therapy, for example, with grandparents and daycare staff.

*One of the disadvantages of home treatment is that all of the responsibility lies with one parent, or two, to manage it [the pump]. It’s a great deal to have to take care of regularly*.

[Health care professional 270]

However, the health care professionals found that letting the parents supervise others was challenging because they worried that training others might put an additional burden on the parent or potentially be problematic regarding quality of care and safety.

### Theme 3. Requesting Digital Features to Ensure Skill Level, Safety, and Quality of Care

#### Subtheme: Access to Online One-Way Communication of Knowledge and Training

Parents suggested that online information, guidelines, and instruction videos would be helpful and should be easily accessible in one place, for example, the hospital’s website or an app, making it more straightforward and quicker to find information and training, which would potentially allow parents to solve technical problems themselves.

*I’ve actually thought of something that could be really nice, being able to watch a short video, if there’s something that’s tricky, instead of having to read about it. Because you can’t be sure that you’ll have the peace of mind to understand it [written information] when you’re in the middle of a crisis*.

[Mother 29]

*Sometimes they call and say that they can’t find it. They’ve lost their information material and manual, so they ask: “What should we do when she gets a fever or becomes flush or?”*

[Health care professional F2]

If information on paper got misplaced or was forgotten, a digital version would always be available. Parents suggested that explanatory instruction videos could be developed to show how to solve the most common problems, for example, removing air bubbles in the infusion set or resolving other practical technical issues related to the pump. The health care professionals supported the idea of having information, guidelines, and instruction videos available online to provide professional reassurance that the parents could find information targeted their needs and skills. They explained that instruction videos could also meet their own needs for maintaining their skill level, along with e-learning. Furthermore, both parents and health care professionals expressed how online material would ensure that the information and skills the parents acquired were aligned with the health care professionals. However, the health care professionals had concerns about how to regularly keep online material and instruction videos updated based on the newest evidence-based practices.

Parents and health care professionals described how online guidelines and instruction videos could help bolster sharing responsibility for managing home infusion therapy for those who did not receive training at the hospital. Parents also expressed that this would be useful for children interested in self-managing their therapy, also because they were already familiar with using digital platforms with videos. Instruction videos also represent a useful tool for those who learn better visually, just as they can be subtitled or dubbed into different languages, improving access to allow more families to receive home infusion therapy.

#### Subtheme: Access to Interactive Communication and Support

Parents also mentioned the benefits of video consultations for receiving guidance in managing the pump and resolving any issues with the pump at home to avoid traveling to the hospital. Video consultations would also provide visual advantages that were absent in ordinary telephone conversations in terms of guidance and preventing misunderstandings.

*A support feature would be very good to have. If you [nurses] had a smartphone available to make video calls to film what the pump says, because my wife panicked a little bit and the communication on the phone with the nurse [at the hospital] was not that great; the nurse and my wife misunderstood one another. So, I just drove him to the hospital*.

[Father 112]

Health care professionals also requested the option of using video consultations to reassure parents that their observations were relevant and to guide them in shared problem-solving. Video consultations could potentially improve their sense of safety and professional control by allowing them to assess the child’s condition and the parent’s management of the therapy.

*You’re sure that the parents know how to change the infusion bag. And if you’re in doubt at home, it’s evening and you call and you can’t be guided on the phone, then it would be nice be able to see it to avoid any misunderstandings*.

[Health care professional 208]

However, the health care professionals had concerns. Even though the child was visible on video, they could not use their clinical judgment based on all their senses compared with face-to-face clinical observations at the hospital. They were uneasy about care becoming too digital.

*I think it’s a good idea [home care], but I also worry about not seeing the children: “How are you doing and how do you look?” Can the parents always assess that? The nursing loses something; it simply becomes too digital. That involves using your eyes, touch, and sense of smell and whatever else we run around doing*.

[Health care professional 133]

Both parents and health care professionals stressed that the work procedures and settings for digital interactions would require aligning expectations. For example, video consultations include specific requirements, such as the need for computers with the right equipment, setting up consultations in a confidential setting, and how to manage scheduling consultations, also in the evening and at night, not to mention having skilled health care professionals available to provide support 24/7.

Parents were also interested in being able to send data to the hospital, such as their child’s blood pressure or pictures of skin changes to reassure the parents and health care professionals regarding the parents’ observations. However, some parents had misgivings about perhaps feeling pressured to provide more data than they could manage. In addition, parents and health care professionals alike were uneasy about when and who was responsible for assessing and reacting to the data at the hospital, and the timespan in which parents could expect to receive an answer. Managing incoming measurements would be required for this to succeed and make all parties feel safer. Furthermore, parents and health care professionals suggested that the parents could also receive notifications to ensure that care tasks like taking their child’s temperature and assessing intravenous access were performed at the right time.

## Discussion

### Principal Findings

We found that parents and health care professionals largely had corresponding needs for safety in home infusion therapy but that their preferences for digital features in a future eHealth intervention to meet those needs were based on different rationalities. It was vital that everyone felt safe about using home infusion therapy, and they suggested various digital features to meet their needs to have consistent knowledge, problem-solving skills, reassurance that the parents could manage the home infusion therapy and their child’s well-being, maintain their skill level, and share responsibility with more than one parent. They emphasized the need for an eHealth intervention that provides additional communication support to existing care to enhance safety, self-management, and quality of care.

### Comparison With Previous Work

A key finding was how home infusion therapy was linked to the extended responsibility of the parents and health care professionals, the former at home without the presence of a nurse, and that latter in terms of having the professional responsibility without being present in the child’s home. We suggest that this extended responsibility generates specific needs and preferences for the digital features a future eHealth intervention should have.

Parents found their responsibilities manageable and felt greater sense of control when they had adequate information and training, along with access to skilled health care professionals when contacting the hospital without these resources, they felt unsure. Two other studies describing the parents’ experiences of insecurity and fear of overlooking something important when their child received home intravenous therapy suggested improvements to decrease worries, such as enhanced preparation, alignment of expectations, and accessible visually based and text-based information [3,34]. Another study also found that parental management of care for their child after surgery at home felt insecure, for example, they were unable to identify postoperative complications [35]. The information they had received was partially incomplete, and there was a gap in support at home, such as some health care professionals lacking the right knowledge to provide support contacted by parents [35]. Even though the parents in our study had some similar experiences, they were generally satisfied overall but would like to see digital features bolster existing support.

Another aspect of the extended responsibility was the health care professionals’ concerns about their ability to clinically assess the child’s condition and to know how the parent was managing home infusion therapy. These concerns challenged their professionalism and ethics. For them, the setup comprised nursing at a distance, causing them to worry about how to ensure quality of care. They suggested that various digital features could bridge this distance and had to correspond to the parents’ needs. Both parents and health care professionals suggested monitoring, such as sending temperature data to the hospital and notifications to parents to remember tasks. These features would help meet their need for reassurance and increase their sense of safety. One study showed that participants valued notifications as reminders for activities to be carried out at home [36], while another study on designing a home monitoring system for children with a medically complex condition showed how parents preferred to track symptoms to identify early changes in their child’s health that could lead to an appropriate intervention [37]. However, parents in our study also worried that they would feel pressured to provide more monitoring and data than they could manage. As a result, adjusting the amount of monitoring and continuously assessing how parents manage at home is important to avoid increased caregiver stress.

The suggestions parents and HCPs provided in our study are comparable with the study by Nkoy et al [37] on the needs of caregivers for designing a home monitoring system for children with a medically complex condition. The parents wanted a mobile health tool that included features to track symptoms and report their child’s symptoms that was user friendly, in addition to having the ability to report interventions provided at home, have direct access to hospital through text messaging, and real-time sharing of data, not to mention the capacity to upload a photo or video for the health care professionals [37]. Two studies in which parents managed nursing care for children after surgery or born prematurely developed a successful eHealth tablet for the aforementioned reasons [17,38]. The parents were given an eHealth tablet before their child was discharged from a highly specialized department and that allowed them chat, make video calls, send photos, write daily reports, and get feedback from health care professionals, which facilitated early detection of complications. These features provided support and easy access to communication with health care professionals, creating a sense of security that the parents highly appreciated [17,38]. Due to similarities with our study, this suggest that a corresponding eHealth intervention could support the families and health care professionals in our study.

However, the health care professionals were also concerned that digital features might exacerbate the distance between them and the family, which is in line with a study on mobile health clinic appointments on parenteral nutrition at home done through a tablet [39]. Parents and health care professionals were very satisfied with how convenient and easy communication was, but the health care professionals reported that the inability to make accurate clinical assessments using the tablet was a significant challenge [39]. Research also shows that another concern is that telemedicine may depersonalize the patient and clinician relationship due to a lack of in-person interaction [11,40]. These key concerns must be taken into consideration when developing an eHealth intervention and will be explored further in the subsequent studies of the development and evaluation of the intervention in this research project.

Another concern was the reservations health care professionals had regarding workflow and setting, for example, how to organize and assess incoming data and chats at all times of day. Furthermore, organizational issues like maintaining confidentiality had to be considered when doing planned and unplanned video consultations. The study with the eHealth tablet showed that parents felt they had to wait too long to receive answers or feedback from health care professionals at the hospital [17]. Another study reported that clinicians emphasized the importance of having clear guidelines for scheduling and use of telemedicine in pediatric emergency settings [11]. Finally, a systematic review on factors concerning the success and failure of eHealth interventions also showed how workflow caused several barriers to success [41]. Accordingly, it is important to consider the needs of health care professionals, as motivating them to use telehealth can otherwise be challenging [42,43].

The extended responsibility that home infusion therapy entails emphasizes the importance of adjusting information and training, in addition to managing the capacity and needs of the individual child and parents. The parents’ mental resources varied during their child’s treatment, and research shows how parents obtain and act differently regarding health-related information concerning their child [35,44]. Both parents and health care professionals suggested that digital features could support the individualized aspects as the parents could, when necessary, repeat information and training, solve problems, and receive guidance. In addition, the parents and health care professionals in our study were preoccupied with sharing their responsibility with others to avoid distorting the amount of caregiving placed on one parent. Studies show that telemedicine can address these issues, by communication in greater alignment with the parents’ own preferences and pace, not to mention that of families who live far from the hospital and wish to avoid the time and expense transportation and parking [17,38,45].

### Strengths and Limitations

This study’s strengths include its participatory design, which involved families and health care professionals at the beginning of the development process. The demographics and experiences of the sample of children with acute or long-term illness is also varied, just as both mothers and fathers were included, not to mention children to some extent. The child was invited to participate with their parent, and 13 children were present at the interview, 2 of whom participated actively. However, separate interviews would improve our understanding of the child’s needs since the parent may withhold experiences and opinions due to the child’s presence. Age-specific methods could have been used to involve and interview children, which would have provided additional insights into their unique needs and preferences. Children will be involved and interviewed in the evaluation study of this research project. The study is conducted in a specific geographic and health care setting, which may limit the generalizability of the findings to other countries and settings with different health care systems.

Data were collected during the early phase of the COVID-19 pandemic, and video consultations and other telehealth options were not used at the department on a regular basis at that time. This may have influenced participants’ perceptions of eHealth and impacted the results in the sense that their technology readiness was limited. Since then, the use of telehealth has increased extensively in Denmark and abroad, perhaps causing our findings to appear obvious.

### Conclusions

Our study highlights the need parents and health care professionals have for increased safety and easier access to communication when receiving and providing home infusion therapy. Their needs and preferences corresponded with each other, and they suggested key digital features for a pediatric eHealth intervention to provide consistent knowledge, skills, and problem-solving during home infusion therapy. The study emphasizes the need to increase access to home infusion therapy by using digital features, and our findings can provide clarity in terms of developing an eHealth intervention to support pediatric home infusion therapy tailored to individual needs.
